# Effects of human herpesvirus 6B reactivation on cognitive function in cord blood transplant recipients: a prospective multicenter study

**DOI:** 10.1007/s12185-024-03714-2

**Published:** 2024-02-26

**Authors:** Masao Ogata, Kumi Oshima, Kuniko Takano, Rie Kawano, Yasunori Ueda, Takashi Imamura, Yukinori Nakamura, Takahiro Okada, Tomomi Toubai, Toshimitsu Ueki, Nobuhiko Uoshima, Hiroyuki Ishida, Akihito Shinohara, Sachiko Seo, Takahiro Fukuda, Masatoshi Inagaki

**Affiliations:** 1 Department of Medical Oncology and Hematology, Faculty of Medicine, Oita University, Hasama-Machi, Yufu, Oita 879-5593 Japan; 2 Research Center for GLOBAL and LOCAL Infectious Diseases, Oita University, Oita, Japan; 3 Division of Hematology and Oncology, Department of Internal Medicine, St. Marianna University School of Medicine, Kanagawa, Japan; 4 Department of Hematology, Oita University Hospital, Oita, Japan; 5 Department of Hematology/Oncology, Kurashiki Central Hospital, Okayama, Japan; 6 Department of Clinical Psychology, Kurashiki Central Hospital, Okayama, Japan; 7 Third Department of Internal Medicine, Yamaguchi University School of Medicine, Yamaguchi, Japan; 8 Department of Hematology, Shimane University Hospital, Shimane, Japan; 9 Division of Hematology and Cell Therapy, Department of Internal Medicine III, Faculty of Medicine, Yamagata University, Yamagata, Japan; 10 Department of Hematology, Nagano Red Cross Hospital, Nagano, Japan; 11 Department of Hematology, Japanese Red Cross Kyoto Daini Hospital, Kyoto, Japan; 12 Department of Pediatrics, Kyoto City Hospital, Kyoto, Japan; 13 Department of Hematology, Tokyo Women’s Medical University, Tokyo, Japan; 14 Department of Hematology and Oncology, Dokkyo Medical University, Tochigi, Japan; 15 Department of Hematopoietic Stem Cell Transplantation, National Cancer Center Hospital, Tokyo, Japan; 16 Department of Psychiatry, Faculty of Medicine, Shimane University, Shimane, Japan

**Keywords:** Human herpesvirus-6B, Cognitive function, Memory function, Cord blood transplantation

## Abstract

This prospective multicenter study aimed to determine the effects of human herpesvirus-6B (HHV-6B) reactivation on central nervous system (CNS) function in cord blood transplant (CBT) recipients. Our focus was to track HHV-6B reactivation and evaluate its association with delirium and cognitive function, specifically in the domains of verbal memory, attention/processing speed, and quality of life (QOL). A cohort of 38 patients participated in this study. Of the 37 patients evaluated, seven (18.9%) developed delirium, with six of these cases emerging after HHV-6B reactivation (median lag, 7 days). Evaluation of verbal memory showed that the final trial score for unrelated words at 70 days after transplantation was significantly lower than that before preconditioning (*P* = 0.004) among patients (*n* = 15) who experienced higher-level HHV-6B reactivation (median or higher maximum plasma HHV-6 DNA load for participating patients). Patients without higher-level reactivation did not show significant declines in verbal memory scores. QOL was assessed using the 36-item Short-Form Health Survey, and the social functioning score 1 year post-transplantation was significantly lower in patients who experienced higher-level HHV-6B reactivation than in those who did not. Our findings suggest that higher-level HHV-6B reactivation can detrimentally affect certain cognitive functions in CBT recipients.

**Supplementary Information:**

The online version contains supplementary material available at 10.1007/s12185-024-03714-2.

## Introduction

Human herpesvirus (HHV)-6B, belonging to the Betaherpesvirinae subfamily, establishes latent infections in the majority of the general population. HHV-6B reactivation after allogeneic hematopoietic stem cell transplantation has been documented in approximately half of bone marrow or peripheral blood stem cell transplant recipients and in 90% of cord blood transplant (CBT) recipients [1, 2]. HHV-6B reactivation is associated with the development of HHV-6B encephalitis, a serious neurological complication post-transplantation. The incidence rates of HHV-6B encephalitis range from 0.5 to 1.2% in bone marrow or peripheral blood stem cell transplant recipients and 8–10% in CBT recipients [2]. Interestingly, despite the prevalent reactivation of HHV-6B, only a fraction of patients are diagnosed with HHV-6B encephalitis. Although most HHV-6B reactivations are deemed asymptomatic, it is conceivable that a robust HHV-6B reactivation may adversely affect the central nervous system (CNS) in a larger patient cohort than that currently diagnosed.

A single-center study by Zerr et al. showed a correlation between HHV-6B reactivation and the onset of delirium and neurocognitive deficits, notably in attention, processing speed, and concentration [3]. Nevertheless, these seminal findings require validation, and the effects of HHV-6B reactivation on long-term survival remain largely unknown.

Considering the frequent HHV-6B reactivation and elevated viral loads in CBT recipients [2, 4], our study aimed to examine the association between HHV-6B reactivation and the onset of delirium and to determine its effects on memory function, concentration, and quality of life (QOL) in CBT recipients. Our overarching goal was to elucidate the broader implications of HHV-6B reactivation on the CNS.

## Patients and methods

### Study design

This prospective, multicenter observational study was organized by the Japan Society for Hematopoietic Cell Transplantation (now known as the Japanese Society for Transplantation and Cellular Therapy). The study protocol was approved by the Ethics Committee of the Japan Society for Hematopoietic Cell Transplantation and the institutional review boards of each participating center. Written informed consent was obtained from all participants in accordance with the Declaration of Helsinki. All procedures strictly adhered to pertinent guidelines and regulations.

### Eligibility criteria

Patients who underwent CBT from December 2018 to December 2020 and were aged ≥ 16 years at the time of transplantation were included in this study. Patients with a HHV-6B monitoring period < 14 days after transplantation, without any type of neuropsychiatric evaluation, or having consistent HHV-6 DNA-positive results with inherited chromosomally integrated HHV-6 were excluded from this study.

### HHV-6B reactivation

Peripheral blood samples were prospectively collected, typically twice a week, from the day of transplantation until either 70 days post-transplantation or the date of discharge, whichever came first. These plasma samples were preserved at −30 °C at each institute and then sent to the Oita University Faculty of Medicine. Quantification of the HHV-6 DNA copy number was conducted using real-time polymerase chain reaction (PCR), as described previously [5]. The sensitivity threshold for HHV-6 DNA detection was at approximately 50 copies/ml of plasma.

### Neuropsychiatric evaluation

#### Delirium

Delirium was evaluated using the Delirium Rating Scale (DRS)-R-98 scale [6]. Assessments were conducted twice weekly 1–49 days post-transplantation and reduced to once weekly 50–70 days post-transplantation. The DRS-R-98 consists of 16 items that address both symptoms and temporal aspects of delirium, yielding a maximum possible score of 46 points. Of these, 13 items specifically gauge the severity of delirium, with higher scores denoting greater severity (up to a maximum of 39 points).

#### Memory function assessment

Verbal memory functioning was assessed using the Standard Verbal Paired Associated Learning (S-PA) test on two occasions: once prior to preconditioning and again 70 days post-transplantation. The S-PA test, developed by the Japan Advanced Brain Dysfunction Society [7], is specifically designed to evaluate linguistic memory [8]. It consists of 10 pairs of words, which can be either semantically related or unrelated. During the assessment, the evaluator, who can be a clinical psychologist or a trained nurse, orally presents a set of 10 word pairs, either related or unrelated. Subsequently, the evaluator articulates the first word from each pair, prompting the patient to orally respond to the corresponding word. The total number of accurate responses is subsequently recorded. This procedure is replicated three times using an identical set of word pairs.

#### Attention and processing speed assessment

Concentration, attention, and processing speed were evaluated using the Symbol Search Subtest of the Wechsler Adult Intelligence Scale-Third Edition (WAIS-III) [9–11] both prior to preconditioning and 70 days post-transplantation. This subtest is a timed pencil-and-paper activity. During the test, patients are tasked with identifying within 120 s whether either of the two target symbols appears in a search group of five adjacent symbols. The final score is derived by subtracting the number of incorrect responses from the number of correct responses.

### Quality of life (QOL) assessment

QOL was assessed using two tools: the 36-item Short-Form Health Survey (SF-36) version 2 and Functional Assessment of Cancer Therapy-Bone Marrow Transplant (FACT-BMT) version 4.0. The SF-36 version 2 is a comprehensive 36-item questionnaire that encompasses domains such as physical functioning, mental health, and social functioning [12, 13]. The FACT-BMT, a 37-item self-report questionnaire, incorporates the 27-item FACT-G, which evaluates four QOL domains: physical, social/family, emotional, and functional well-being. Additionally, it includes the BMTS, which is a 10-item subscale that addresses concerns specific to transplantation [14]. The FACT-BMT trial outcome index is computed by summing the scores of the physical and functional well-being sections with the BMTS scores. Both the SF-36 and FACT-BMT were administered to patients at two distinct time points: 70 days and 1 year post-transplantation.

### Definitions

HHV-6B reactivation was defined as the detection of measurable levels of HHV-6 DNA in the plasma. Higher-level HHV-6B reactivation was defined as the reactivation in which HHV-6 DNA reached or exceeded the median of the peak plasma HHV-6 DNA load observed among the participants. Delirium episodes were identified based on a DRS-R98 severity score ≥ 10 points or a total score of ≥ 14.5 points [15]. Acute and chronic graft-versus-host diseases (GVHD) were diagnosed and graded based on established clinical criteria [16, 17]. The early stages were categorized as acute leukemia in the first or second remission, chronic myeloid leukemia in the first chronic phase, or myelodysplastic syndrome without blast excess. Any stage beyond these specifications was defined as a non-early stage. Dosages defined as reduced-intensity conditioning (RIC) regimens were total body irradiation (TBI) ≤ 5 Gy in a single fraction or 5–8 Gy in multiple fractions, busulfan ≤ 8 mg/kg (intravenous busulfan ≤ 6.4 mg/kg), or melphalan ≤ 140 mg/m^2^. Inherited chromosomally integrated HHV-6 was defined as an HHV-6 plasma DNA concentration of ≥ 100 copies/ml that persisted in ≥ 80% of plasma samples.

### Statistical considerations

The probability of the first incidence of HHV-6B reactivation was calculated based on cumulative incidence curves according to the method described by Fine and Gray [18]. The discontinuation of HHV-6 DNA monitoring was considered a competing event. The effects of factors on the onset of delirium were examined through univariate analysis using Fisher’s exact tests. Alterations observed in the S-PA, Symbol Search Subtest, and SF-36 scores over time were analyzed using paired t-tests. The SF-36 and FACT-BMT scores according to the study variables were compared using the Mann–Whitney *U* test. All statistical tests were two sided, and the significant level was set at 5%. The analyses were performed using EZR [19] version 1.32 (Saitama Medical Center, Jichi Medical University), which is a graphical user interface for R version 2.13.0 (The R Foundation for Statistical Computing, Vienna, Austria).

## Results

### Patient characteristics

Forty-five patients were enrolled from December 2018 to December 2020. Of these, 38 patients were included in this study. Seven patients were excluded because of the following reasons: no neuropsychiatric evaluation (*n* = 5), HHV-6B monitoring < 14 days after transplantation (*n* = 1), and duplicate registration (*n* = 1). The characteristics of patients included in the analyses are summarized in Table 1.

**Table 1:** Patients’ characteristics

Characteristics	N (%)
Age at transplantation, years	
Median (range)	57 (20–70)
16–54 years	17 (45)
≥ 55 years	21 (55)
Male sex	19 (50)
Underlying disease	
Acute myeloid leukemia	15 (39)
Myelodysplastic syndrome	5 (13)
Chronic myeloid leukemia	1 (2.6)
T-cell large granular lymphocytic leukemia	1 (2.6)
Adult T-cell leukemia	1 (2.6)
Malignant lymphoma	6 (16)
Multiple myeloma	1 (2.6)
Disease phase at transplantation	
Early	17 (45)
Non-early	21 (55)
Preconditioning regimen	
MAC	21 (55)
RIC	17 (45)
TBI in preconditioning	
0	13 (34)
1–8 Gy	15 (39)
> 8 Gy	10 (26)
HLA disparities	
0	2 (5.3)
1	10 (26)
2	26 (68)
CNS involvement of underlying disease	
None	36 (95)
History	2 (5.3)
At the time of transplantation	0 (0)
Mental illness	
History	1 (2.6)^a^
At the start of preconditioning	1 (2.6)^b^

*MAC* myeloablative conditioning, *RIC* reduced-intensity conditioning, *TBI* total body irradiation, *HLA* human leukocyte antigen, *CNS* central nervous system

^a^Depression

^b^Adjustment disorder

### HHV-6B reactivation and HHV-6B encephalitis

In total, 674 plasma samples collected 70 days after transplantation were examined using real-time PCR. Blood sample collection began at a median of 3 (range, 1–7) days after transplantation and lasted for a median of 68 (range, 19–70) days after transplantation. The median number of blood samples per patient was 19 (range, 6–21).

**Fig. 1:**
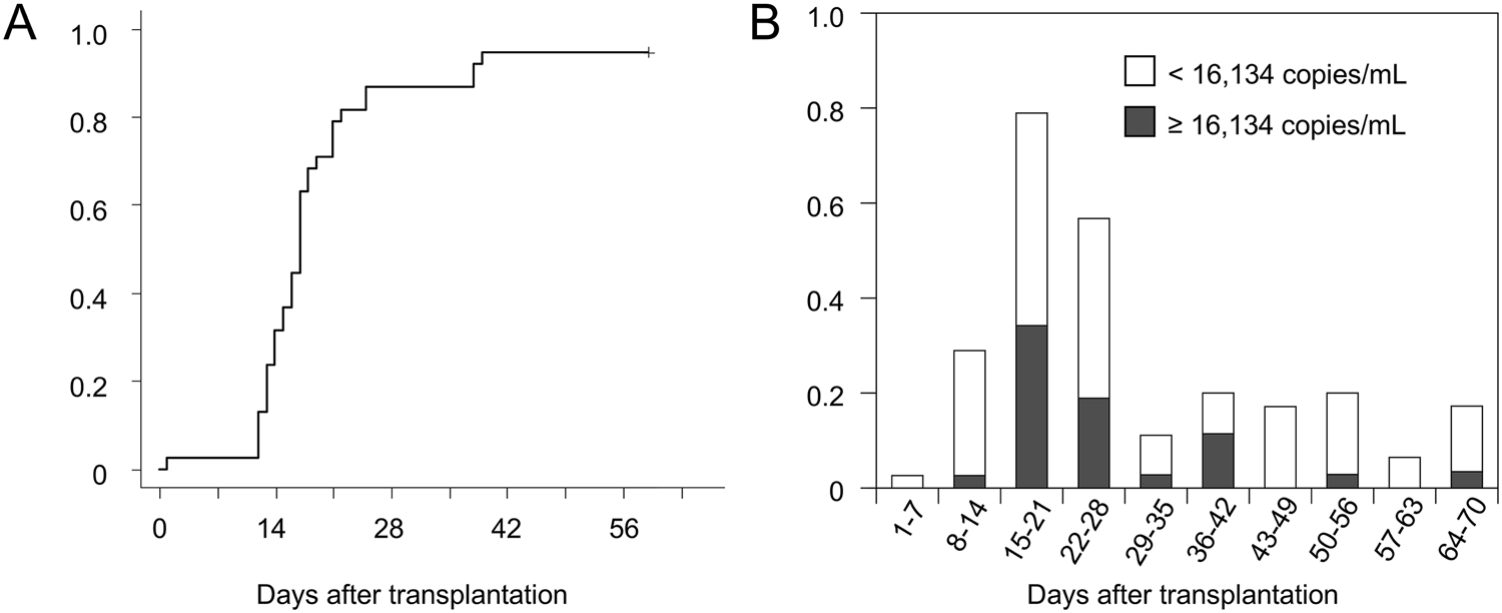
**A** Cumulative incidence of the first detection of plasma human herpesvirus (HHV)-6B. **B** Proportion of patients with positive HHV-6 DNA results in each post-transplantation period. In this study, the median value of the maximum plasma HHV-6 load in each patient was 16,134 copies/ml. Gray bars indicate the rate of patients who displayed plasma HHV-6 DNA at ≥ 16,134 copies/ml

During the observation period, 36 (94.7%) of the 38 patients tested positive for plasma HHV-6 DNA at least once. The cumulative incidence rate of the first detection of HHV-6B reactivation 60 days after transplantation was 94.7% (95% confidence interval, 76.2%–98.9%) (Fig. 1A). HHV-6B reactivation was frequently apparent in the plasma 15–28 days (3rd and 4th weeks) after transplantation (Fig. 1B). The median peak plasma HHV-6 DNA load in the participating patients was 16,134 copies/ml (range, less than the detection limit to 270,800 copies/ml). Consequently, we set the threshold for “higher-level HHV-6B reactivation” at plasma HHV-6 DNA loads of ≥ 16,134 copies/ml. Among the 38 patients, 19 exhibited higher-level HHV-6B reactivation, whereas the other 19 did not.

Within 70 days following transplantation, HHV-6B encephalitis was observed in 5 of the 38 patients, accounting for 13.2% of the cohort. The cumulative incidence rate of HHV-6B encephalitis 70 days after transplantation was 13.5% (95% confidence interval, 1.8%–23.9%). The median (range) plasma HHV-6 DNA copy number at the onset of HHV-6B encephalitis in these five patients was 2,844 copies/ml (range, 1768–143,800 copies/ml).

### Delirium assessment

**Fig. 2:**
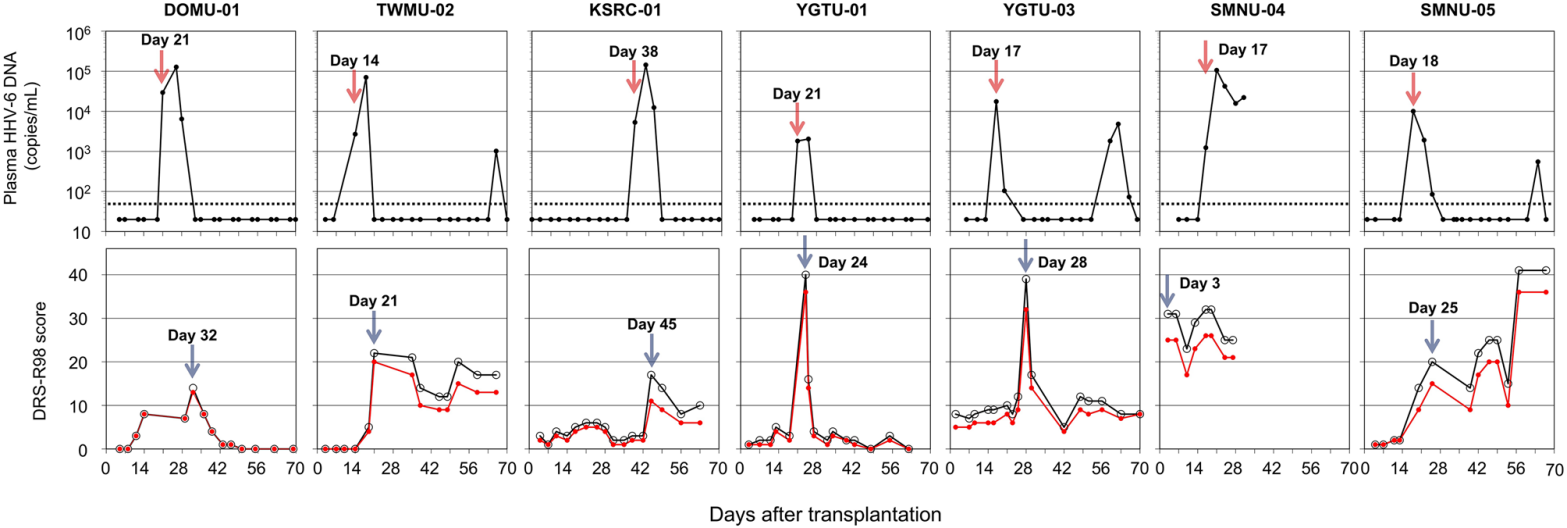
Kinetics of HHV-6 DNA copy number (upper row) and change of Delirium Rating Scale (DRS)-R scores (bottom row) for seven patients who developed delirium. In the upper row, the dashed line indicates the threshold for HHV-6 DNA detection. The arrow and denoted day indicate the first day of confirmed plasma HHV-6 DNA positivity. In the bottom row, the black and red lines show the DRS-R total and severity scores, respectively. The arrow and denoted day indicate the first day delirium was diagnosed

Of the 38 patients, 37 were evaluated for delirium using the DRS-R-98. One patient opted out of the delirium assessment. The median number of DRS-R-98 assessments per patient during the observation period of 70 days post-transplantation was 16 (range, 3–19). Seven (18.9%) patients developed delirium. Figure 2 shows the kinetics of plasma HHV-6 DNA and the DRS-R-98 total and severity scores in patients with delirium. One patient (SMNU-04) had already developed delirium 3 days after transplantation. In the remaining six patients, delirium developed after HHV-6 DNA in the plasma displayed positive results, with a median lag of 7 (range, 3–11 days) days. Univariate analysis showed that age ≥ 55 years was significantly associated with the development of delirium (*P* = 0.012), but other variables, including disease status (early vs*.* non-early), preconditioning [myeloablative conditioning (MAC) vs. RIC], TBI (< 8 Gy vs. ≥ 8 Gy), acute GVHD (< grade II vs. ≥ grade II), and peak HHV-6 DNA (higher level *vs.* other than higher level), were not identified as risk factors for developing delirium (Supplementary Table 1).

Among the five patients diagnosed with HHV-6B encephalitis by their attending physicians, two (KSRC-01 and YGTU-01) were also assessed to have developed delirium concurrent with the onset of HHV-6B encephalitis. In contrast, the remaining three patients did not exhibit signs of delirium (Supplementary Figure 1). In these three patients, HHV-6B encephalitis was diagnosed when subtle CNS symptoms became evident, prompting timely antiviral interventions.

### Memory function

Of the initial cohort of 38 participants, 24 underwent S-PA assessments at two points: prior to preconditioning and at 70 days post-transplantation. The other 14 patients missed S-PA evaluations for various reasons: clinical deterioration or death (*n* = 6), unavailability of a clinical psychologist (*n* = 3), patient refusal (*n* = 2), administrative oversights (*n* = 2), or unspecified reasons (*n* = 1). Two patients who developed delirium and three patients who were diagnosed with HHV-6B encephalitis underwent S-PA testing.

**Fig. 3:**
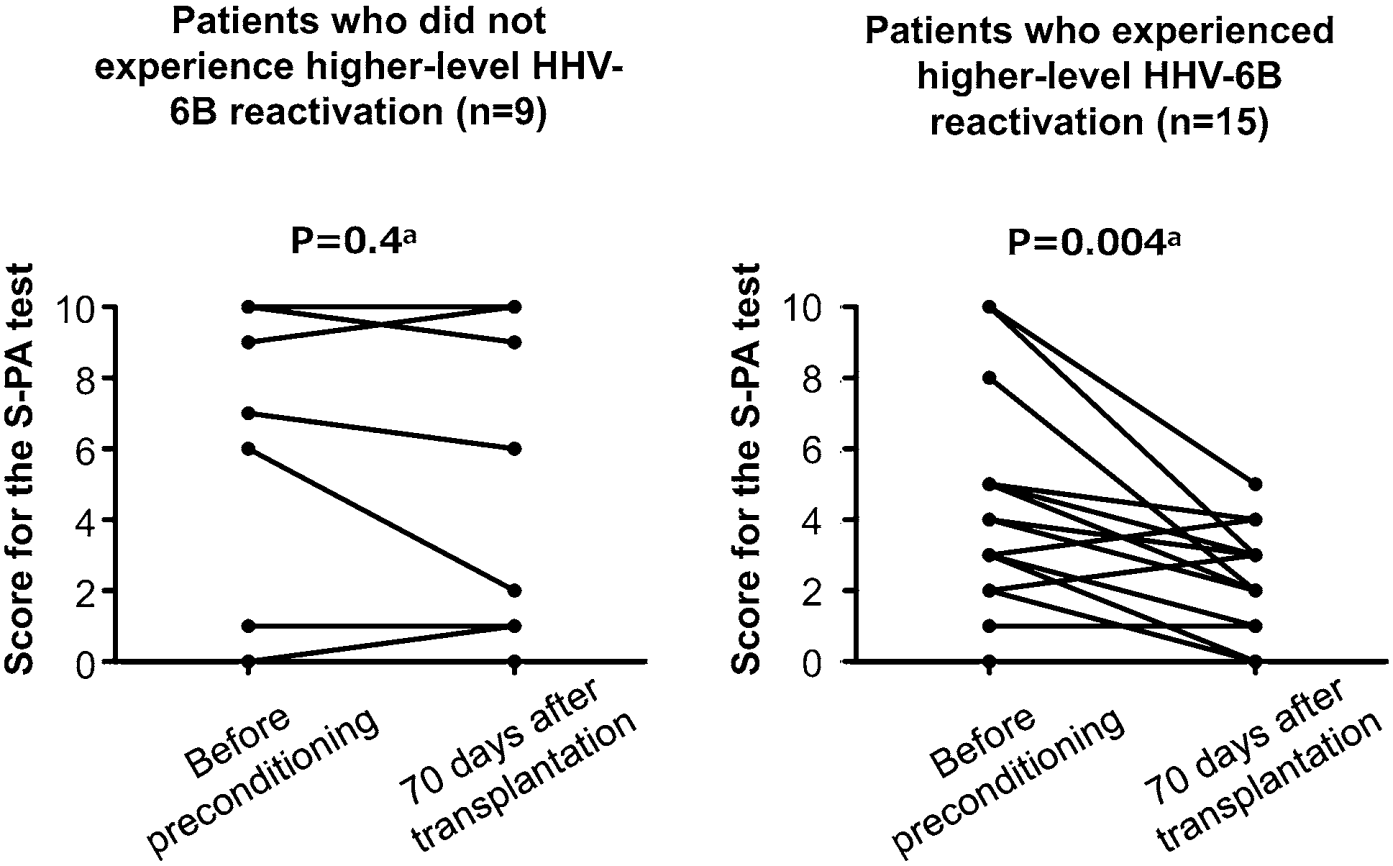
The changes in the final trial scores on the Standard Verbal Paired Associated Learning (S-PA) test for unrelated words before preconditioning and at 70 days after transplantation. Higher-level reactivation was defined as reactivation of HHV-6 DNA ≥ 16,134 copies/mL (the median value of the maximum plasma HHV-6 load in participating patients). ^a^Paired *t*-test

Table 2 shows the S-PA scores in the final trial score for unrelated words obtained before preconditioning and 70 days after transplantation. A significant decline in scores was observed in patients with female gender, early disease status, MAC preconditioning, TBI < 8 Gy, and higher-level HHV-6B reactivation but not in patients with male gender, non-early disease status, RIC preconditioning, with TBI ≥ 8 Gy, and no higher-level HHV-6B reactivation. The changes in the final trial scores for unrelated words in patients who experienced higher-level HHV-6B reactivation and those who did not are illustrated in Fig. 3. Regarding the patient background in this subset, age, sex, disease status, and preconditioning regimen showed no significant differences between patients who experienced higher-level reactivation and those who did not. However, the incidence rate of acute GVHD was significantly higher in patients with higher-level HHV-6 reactivation than in those without higher-level HHV-6B reactivation (Table 3). A similar trend was observed in the analysis that excluded three patients diagnosed with HHV-6B encephalitis (Supplementary Table 2).

**Table 2:** Scores for the standard verbal paired associate leaning (S-PA) tests

Variables	Unrelated words, score for final (third) trial, mean (SD)		*P*^a^
	Before the start of preconditioning	70 days after transplantation	
Total cases (*N* = 24)	4.9 (3.6)	3.4 (3.3)	0.003
Age, years			
< 55 (*n* = 13)	5.8 (3.6)	4.4 (3.8)	0.041
≥ 55 (*n* = 11)	3.8 (3.4)	2.3 (2.1)	0.046
Gender			
Male (*n* = 13)	2.8 (2.8)	2.3 (2.7)	0.17
Female (*n* = 11)	7.4 (2.8)	4.7 (3.6)	0.007
Disease status at transplantation			
Early (*n* = 13)	5.7 (3.9)	4 (3.8)	0.027
Non-early (*n* = 11)	4 (3.1)	2.7 (2.6)	0.067
Preconditioning			
MAC (*n* = 15)	5.8 (3.7)	4.2 (3.7)	0.02
RIC (*n* = 9)	3.4 (3.0)	2.1 (2.1)	0.096
TBI			
≤ 8 Gy (*n* = 15)	4.7 (3.6)	3.3 (3.1)	0.012
> 8 Gy (*n* = 9)	5.2 (3.7)	3.7 (3.7)	0.13
Acute GVHD			
< Grade II (*n* = 14)	6.2 (3.8)	4.6 (3.8)	0.033
≥ Grade II (*n* = 10)	3.1 (2.3)	1.7 (1.3)	0.0498
HHV-6B reactivation^b^			
Not higher-level reactivation (*n* = 9)	5.9 (4.4)	5.4 (4.4)	0.4
Higher-level reactivation (*n* = 15)	4.3 (3.0)	2.2 (1.6)	0.004

*SD* standard deviation, *MAC* myeloablative conditioning, *RIC* reduced-intensity conditioning, *TBI* total body irradiation, *GVHD* graft-versus-host disease, *HHV-6B* human herpesvirus 6B

^a^Paired *t*-test

^b^Higher-level HHV-6B reactivation was defined as plasma HHV-6 DNA ≥ 16 134 copies/mL, which was the median value of maximum plasma HHV-6 load in participating patients

**Table 3:** Comparison of patients with and without higher-level HHV-6B reactivation

Variables	Patients who did not experience high-level HHV-6B reactivation (*n* = 9)	Patients who experienced higher-level HHV-6B reactivation (*n* = 15)	*P*^a^
Peak HHV-6B DNA (copies/mL), median (range)	4547 (1063–14,200)	40,000 (16,500–270,800)	< 0.001
Age in years, median (range)	44 (30–64)	57 (22–70)	0.50
Age in years, ≥ 55 years	3 (33)	8 (53)	0.42
Male sex	4 (44)	9 (60)	0.68
Non-early disease status at transplantation	3 (33)	8 (53)	0.42
MAC preconditioning	7 (78)	8 (53)	0.39
TBI > 8 Gy	4 (44)	5 (33)	0.68
Acute GVHD ≥ Grade II	1 (11)	9 (60)	0.03

Data are number (%), unless otherwise indicated

*MAC* myeloablative conditioning, *TBI* total body irradiation, *GVHD* graft-versus-host disease, *HHV-6B* human herpesvirus 6B

^a^Fisher’s exact test

As the mean score for unrelated words in all patients decreased from 4.9 to 3.4 (indicating a 1.5-point decrease), we further determined the factors associated with score reductions of ≥ 2 points (Table 4). The univariate analysis showed that only higher-level HHV-6B reactivation was significantly associated with a score drop of ≥ 2 points.

**Table 4:** Factors associated with a decrease of ≥ 2 points on the third Standard Verbal Paired Associate Leaning test for unrelated language (univariate analysis)

Variables	*N* (%)	*P*^a^
Age, years		
< 55 (*n* = 13)	6 (46)	0.70
≥ 55 (*n* = 11)	4 (36)	
Gender		
Male (*n* = 13)	4 (31)	0.41
Female (*n* = 11)	6 (54)	
Disease status at transplantation		
Early (*n* = 13)	5 (38)	1.00
Non-early (*n* = 11)	5 (45)	
Preconditioning		
MAC (*n* = 15)	7 (47)	0.68
RIC (*n* = 9)	3 (33)	
TBI		
≤ 8 Gy (*n* = 15)	6 (40)	1.00
> 8 Gy (*n* = 9)	4 (44)	
Acute GVHD		
< Grade II (*n* = 14)	5 (36)	0.68
≥ Grade II (*n* = 10)	5 (50)	
HHV-6B reactivation		
Not higher-level reactivation (*n* = 9)	1 (11)	0.033
Higher-level reactivation (*n* = 15)	9 (60)	

*MAC* myeloablative conditioning, *RIC* reduced-intensity conditioning, *TBI* total body irradiation, *GVHD* graft-versus-host disease, *HHV-6B* human herpesvirus 6B

^a^ Fisher’s exact test

### Attention and processing speed

Of the 38 participants, 22 underwent the WAIS-III Symbol Search Subtest at two points: prior to preconditioning and at 70 days post-transplantation. The remaining 16 participants did not complete the subtest for various reasons: clinical deterioration or death (*n* = 6), unavailability of a clinical psychologist (*n* = 3), patient refusal (*n* = 2), administrative oversight (*n* = 2), visual impairment preventing test completion (*n* = 1), or unspecified reasons (*n* = 2). One patient who developed delirium and two who were diagnosed with HHV-6B encephalitis underwent testing.

The Symbol Search Subtest scores did not significantly change from before preconditioning to 70 days after transplantation, and no variables, including disease status, preconditioning, TBI, acute GVHD, and peak HHV-6 DNA, were associated with changes in the scores (Supplementary Table 3).

### QOL

Of the 38 participants, 19 completed the SF-36 version 2 at 70 days and 1 year post-transplantation. The remaining 19 participants were unable to complete the SF-36 for various reasons, including death (*n* = 11), clinical deterioration (*n* = 1), administrative oversight (*n* = 4), transfer to another hospital (*n* = 1), patient refusal (*n* = 1), and unspecified reasons (*n* = 1). Three patients who were diagnosed with HHV-6B encephalitis underwent SF-36 testing.

Table 5 presents the SF-36 scores. One year post-transplantation, the role/social component summary was significantly lower in patients aged ≥ 55 years and those who experienced higher-level HHV-6B reactivation than in younger patients and those who did not experience higher-level HHV-6B reactivation. Patients who had not experienced higher-level HHV-6B reactivation (*n* = 7) showed a significant improvement in this score between 70 days and 1 year after transplantation (mean score, 29.5 and 46.5, respectively; *P* = 0.031; paired *t*-test). Conversely, no improvement was observed in patients who experienced higher-level HHV-6B reactivation (*n* = 12) (mean scores, 33.8 and 32.5, respectively; *P* = 0.68; paired *t*-test).

**Table 5:** Scores for the Short-Form Health Survey (SF-36)

	Physical component summary				Mental component summary				Role/social component summary			
	Day 70	*P*^a^	1 year	*P*^a^	Day 70	*P*^a^	1 year	*P*^a^	Day 70	*P*^a^	1 year	*P*^a^
	Mean (SD)		Mean (SD)		Mean (SD)		Mean (SD)		Mean (SD)		Mean (SD)	
Total cases (*N* = 19)	36.2 (11.1)		45.3 (12.9)		56.1 (5.8)		54.3 (10.6)		32.3 (14.0)		37.7 (12.1)	
Age, years												
< 55 (*n* = 11)	35.2 (8.3)	0.71	44.1 (7.4)	0.62	56.8 (6.8)	0.59	56.9 (8.6)	0.84	34.0 (14.5)	0.77	43.6 (9.4)	0.023
≥ 55 (*n* = 8)	37.5 (14.7)		44.2 (18.6)		55.2 (4.3)		53.6 (13.5)		30.7 (14.2)		30.6 (12.4)	
Acute GVHD												
< Grade II (*n* = 13)	35.9 (12.3)	0.63	47 (11.5)	0.69	56.7 (6.8)	0.69	53.2 (12.6)	0.97	28.9 (12.0)	0.17	40.4 (12.1)	0.14
≥ Grade II (*n* = 6)	36.8 (9.1)		41.7 (16.1)		55.0 (2.8)		56.7 (4.3)		39.4 (16.5)		31.6 (10.5)	
Chronic GVHD												
None (*n* = 10)	34.3 (10.8)	0.51	39.6 (14.2)	0.034	56.8 (4.4)	0.51	56.2 (10.5)	0.35	32.7 (12.4)	0.97	36.6 (14.7)	0.74
Limited or extensive (*n* = 9)	38.2 (11.8)		51.6 (7.9)		55.4 (7.3)		52.3 (11.0)		31.8 (16.4)		38.9 (9.2)	
Relapse of underlying disease within 1 year after transplantation												
No (*n* = 15)	36.9 (12.1)	0.58	45.6 (14.4)	0.45	55.8 (5.9)	0.65	55.1 (9.4)	0.58	32.3 (13.1)	0.65	39.7 (10.4)	0.29
Yes (*n* = 4)	33.5 (6.9)		44.2 (4.6)		57.6 (6.2)		51.3 (15.8)		32.2 (19.5)		30.0 (16.6)	
HHV-6B reactivation												
Not higher-level reactivation (*n* = 7)	34.9 (10.5)	0.74	51.0 (8.4)	0.15	58.9 (7.7)	0.15	52.1 (12.5)	0.64	29.5 (14.7)	0.53	46.5 (9.0)	0.008
Higher-level reactivation (*n* = 12)	36.9 (11.9)		42.0 (14.1)		54.5 (3.9)		55.7 (9.7)		33.8 (14.1)		32.5 (10.8)	

*SD* standard deviation, *GVHD* graft-versus-host disease, *HHV-6B* human herpesvirus 6B

^a^Mann–Whitney *U* test

Additionally, 19 patients completed the FACT-BMT testing at both the 70-day and 1-year post-transplantation. No variables were associated with the FACT-BMT trial outcome score, FACT-G total score, or FACT-BMT total score (Supplementary Table 4).

## Discussion

The kinetics of HHV-6B reactivation after allogeneic stem cell transplantation is unique. Typically, patients experience short-term reactivation near neutrophil engraftment [5]. Occasionally, a rapid increase in plasma HHV-6 DNA levels can be detected. Such spikes in HHV-6B often precede the onset of HHV-6B encephalitis [2, 5], suggesting that higher-level HHV-6B reactivation can inflict damage to the CNS within a short span. We hypothesized that even in patients who were not diagnosed with HHV-6B encephalitis, significant HHV-6B reactivation might exert some effects on CNS functioning.

Delirium episodes were identified in seven patients through mental status monitoring. In six of these patients, delirium manifested for roughly 1 week (ranging between 3 and 11 days) after the detection of HHV-6 DNA in plasma. Zerr et al. have reported a temporal association between delirium episodes and HHV-6B reactivation during allogeneic stem cell transplantation [3], and Hill et al. have reported a quantitative association between HHV-6 reactivation and delirium in CBT recipients [20]. Our findings are consistent with these observations. Although the causal relationship between HHV-6B reactivation and delirium has not been well established currently [21], HHV-6B-associated delirium may be more common than previously recognized.

In the third trial of the recall of unrelated words, patients who experienced a higher-level HHV-6B reactivation showed a significantly lower score on day 70 after transplantation compared with their score before preconditioning. In contrast, those who did not experience higher-level HHV-6B reactivation showed no significant decline in their scores. Univariate analysis further showed a significant association between higher-level HHV-6B reactivation and a decline of ≥ 2 points in the unrelated word recall score. These results suggest that higher-level HHV-6B reactivation can influence verbal memory in CBT recipients. A significant reduction in S-PA scores was noted in the MAC group; however, this trend was not observed in the TBI > 8 Gy group. Given the similarity in mean score reductions between the TBI ≤ 8 Gy and > 8 Gy groups, the lack of a significant decline in the TBI > 8 Gy group may be attributed to its smaller sample size.

No significant decrease was observed when comparing the scores for attention/processing speed from pre-preconditioning to 70 days post-transplantation, even in patients with higher-level HHV-6B reactivation. Although declines in memory scores are occasionally associated with reductions in attention, our data indicate that the observed decrease in the verbal memory domains is not due to deficits in the attention/processing domain.

Zerr et al. found that HHV-6B reactivation was linked to deficits in attention and processing speed but not memory [3]. In contrast, our study, focusing solely on cord blood transplant recipients, indicated an association between higher-level HHV-6B reactivation (≥ 16,134 copies/mL) and memory impairment. This contrast may stem from differences in study designs and patient populations. Zerr et al.’s median HHV-6 DNA level was lower (873 copies/mL, interquartile range 175–4580 copies/mL), suggesting fewer cases with high reactivation. Additionally, their approach involved comparing proportions of cases with declines greater than 0.75 standard deviations, a method differing from our use of paired *t*-tests for overall score comparison. These methodological variations could explain the different attention processing speed deficits.

Evaluation of QOL assessed using the SF-36 revealed that at 1 year post-transplantation, social functioning was significantly lower in patients who experienced higher-level HHV-6B reactivation than in other patients. Notably, although social functioning significantly improved between 70 days and 1 year after transplantation in patients who did not experience higher-level HHV-6B reactivation, no analogous improvement was observed in those who experienced higher-level HHV-6B reactivation. The underlying reason for this observation remains unclear. However, higher-level HHV-6B reactivation may compromise functions vital for fulfilling social roles, such as memory.

A significant limitation of our study was the small number of patients examined. Acquiring a large sample size proved challenging because few facilities offered neurological evaluation by specialists. Moreover, the attending physicians often deemed the examination considerably taxing for patients who experienced transplant complications. Furthermore, many patients either deteriorated or died before undergoing QOL and cognitive function assessments. Due to the limited number of patients, multivariate analysis was not possible; factors other than HHV-6B may be associated with both viral reactivation and brain damage; however, this was difficult to assess in the present study.

Although our data may not be definitive, considering the limited number of patients analyzed, our findings offer a novel perspective suggesting that HHV-6B reactivation is associated with the subsequent onset of delirium, a decline in verbal memory score, and diminished social functioning. These observations highlight the potential benefits of adopting a preventative strategy against HHV-6B reactivation to preserve cognitive function and QOL in CBT recipients. To generalize these conclusions, validating them in a larger patient cohort is crucial.

### Supplementary Information

Below is the link to the electronic supplementary material.

Supplementary file1 (DOCX 153 KB)

Supplementary file2 (DOCX 20 KB)

Supplementary file3 (DOCX 22 KB)

Supplementary file4 (DOCX 20 KB)

Supplementary file5 (DOCX 25 KB)

## Data Availability

Data supporting the findings of this study are available from the corresponding author upon reasonable request. The data are not publicly available due to privacy or ethical restrictions.
