# Supplementary material for: Effects of human herpesvirus 6B reactivation on cognitive function in cord blood transplant recipients: a prospective multicenter study
Source: Int J Hematol. 2024 Feb 26;119(4):432–41. doi: 10.1007/s12185-024-03714-2 (PMC10960775; doi:10.1007/s12185-024-03714-2)
Supplement: Supplementary file 1 — Supplementary file1 (DOCX 153 KB) [file 12185_2024_3714_MOESM1_ESM.docx]

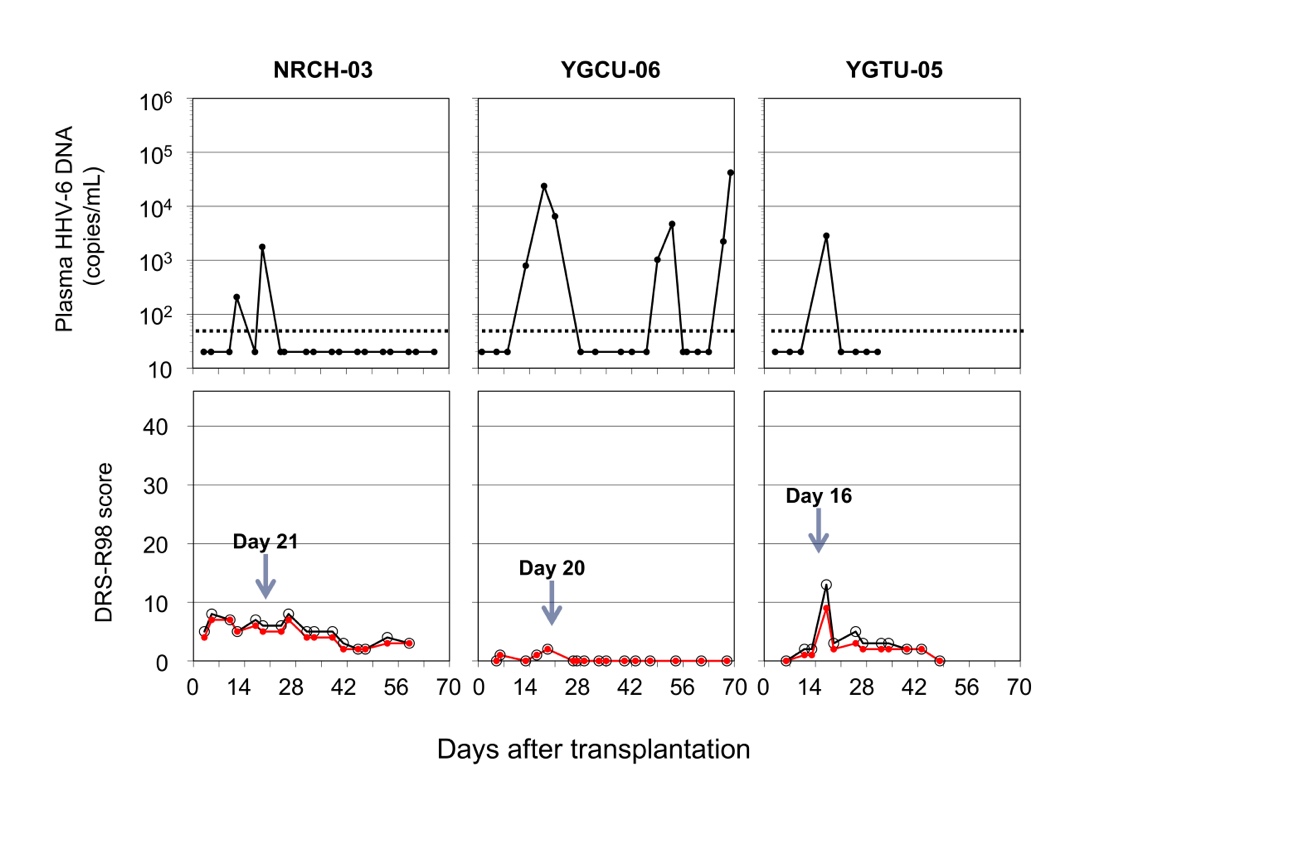


**Supplementary Fig. 1.**

Kinetics of HHV-6 DNA copy number (upper row) and change of DRS-R score (bottom row) in 3 patients who did not develop delirium but were determined to develop HHV-6B encephalitis. In the upper row, the dashed line indicates the threshold for HHV-6 DNA detection. In the bottom row, the black and red lines show the DRS-R total and severity scores, respectively. The arrow and denoted day indicate the day HHV-6B encephalitis diagnosed.
