# Supplementary material for: Effects of human herpesvirus 6B reactivation on cognitive function in cord blood transplant recipients: a prospective multicenter study
Source: Int J Hematol. 2024 Feb 26;119(4):432–41. doi: 10.1007/s12185-024-03714-2 (PMC10960775; doi:10.1007/s12185-024-03714-2)
Supplement: Supplementary file 2 — Supplementary file2 (DOCX 20 KB) [file 12185_2024_3714_MOESM2_ESM.docx]

**Supplementary Table 1. Incidence of delirium according to study variables**

| **Variables** | **Incidence of delirium**  **Up to day 70 (%)** | ***P*^a^** |
| --- | --- | --- |
| **Age, years** |  |  |
| <55 (n=16) | 0 | 0.012 |
| ≥55 (n=21) | 33.3 |  |
| **Disease status at transplantation** |  |  |
| Early (n=17) | 11.8 | 0.42 |
| Non-early (n=20) | 25 |  |
| **Preconditioning** |  |  |
| MAC (n=19) | 10.5 | 0.23 |
| RIC (n=18) | 27.8 |  |
| **TBI** |  |  |
| ≤8 Gy (n=28) | 25 | 0.16 |
| > 8Gy (n=9) | 0 |  |
| **Acute GVHD** |  |  |
| < Grade II (n=24) | 20.8 | 1 |
| ≥ Grade II (n=13) | 15.4 |  |
| **HHV-6B reactivation^b^** |  |  |
| Not higher-level reactivation (n=19) | 10.5 | 0.23 |
| Higher-level reactivation (n=18) | 27.8 |  |

*MAC* myeloablative conditioning, *RIC* reduced-intensity conditioning, *TBI* total body irradiation, *GVHD* graft versus host disease, *HHV-6B* human herpesvirus 6B.

^a^ Fisher’s exact test.

^b^ High level HHV-6 reactivation was defined as plasma HHV-6 DNA ≥16,134 copies/mL, which is the median value of maximum plasma HHV-6 load in each participating patient.
