# Supplementary material for: Effects of human herpesvirus 6B reactivation on cognitive function in cord blood transplant recipients: a prospective multicenter study
Source: Int J Hematol. 2024 Feb 26;119(4):432–41. doi: 10.1007/s12185-024-03714-2 (PMC10960775; doi:10.1007/s12185-024-03714-2)
Supplement: Supplementary file 3 — Supplementary file3 (DOCX 22 KB) [file 12185_2024_3714_MOESM3_ESM.docx]

**Supplementary Table 2. Scores for the Standard Verbal Paired Associate Leaning (S-PA) tests, excluding patients who developed HHV-6B encephalitis**

| **Variables** |  | **Unrelated words, score for final (third) trial, mean (SD)** | |  |
| --- | --- | --- | --- | --- |
|  |  | **Before preconditioning** | **70 days after transplantation** | ***P*^a^** |
| **Total cases** (N=21) |  | 4.3 (3.4) | 3.0 (3.1) | 0.006 |
| **Age, years** |  |  |  |  |
| <55 (n=11) |  | 5.1 (3.3) | 4.0 (3.8) | 0.059 |
| ≥55 (n=10) |  | 3.5 (3.4) | 1.9 (1.9) | 0.061 |
| **Gender** |  |  |  |  |
| Male (n=12) |  | 2.3 (1.9) | 1.7 (1.4) | 0.17 |
| Female (n=9) |  | 7.1 (2.9) | 4.8 (4.0) | 0.017 |
| **Disease status at transplantation** |  |  |  |  |
| Early (n=10) |  | 4.7 (3.9) | 3.3 (3.7) | 0.061 |
| Non-early (n=11) |  | 4.0 (3.1) | 2.7 (2.6) | 0.067 |
| **Preconditioning** |  |  |  |  |
| MAC (n=13) |  | 5.2 (3.6) | 3.8 (3.6) | 0.031 |
| RIC (n=8) |  | 3.0 (2.8) | 1.6 (1.7) | 0.13 |
| **TBI** |  |  |  |  |
| ≤8 Gy (n=14) |  | 4.6 (3.7) | 3.1 (3.1) | 0.016 |
| >8Gy (n=7) |  | 3.9 (2.9) | 2.9 (3.3) | 0.25 |
| **Acute GVHD** |  |  |  |  |
| < Grade II (n=11) |  | 5.4 (3.9) | 4.2 (3.8) | 0.077 |
| ≥ Grade II (n=10) |  | 3.1 (2.3) | 1.7 (1.3) | 0.0498 |
| **HHV-6B reactivation** |  |  |  |  |
| Not higher-level reactivation (n=7) |  | 5.1 (4.7) | 4.7 (4.7) | 0.53 |
| Higher-level reactivation (n=14) |  | 3.9 (2.6) | 2.1 (1.6) | 0.006 |

*HHV-6B* human herpesvirus 6B*, SD* standard deviation, *MAC* myeloablative conditioning, *RIC* reduced-intensity conditioning*, TBI* total body irradiation, *GVHD* graft versus host disease, *HHV-6B* human herpesvirus 6B.

^a^ paired t-test
