# Supplementary material for: Effects of human herpesvirus 6B reactivation on cognitive function in cord blood transplant recipients: a prospective multicenter study
Source: Int J Hematol. 2024 Feb 26;119(4):432–41. doi: 10.1007/s12185-024-03714-2 (PMC10960775; doi:10.1007/s12185-024-03714-2)
Supplement: Supplementary file 4 — Supplementary file4 (DOCX 20 KB) [file 12185_2024_3714_MOESM4_ESM.docx]

**Supplementary Table 3. Scores for the Symbol Search Subtest of the Wechsler Adult Intelligence Scale -Third Edition**

| **Variables** | **Before preconditioning, mean (SD)** | **Day 70 after transplantation, mean (SD)** | ***P*^a^** |
| --- | --- | --- | --- |
| **Total cases** (N=22) | 35.9 (9.5) | 35.2 (10.2) | 0.67 |
| **Age, years** |  |  |  |
| <55 (n=14) | 37.0 (1.4) | 36.0 (10.4) | 0.64 |
| ≥55 (n=8) | 33.9 (10.0) | 33.8 (10.3) | 0.96 |
| **Disease status at transplantation** |  |  |  |
| Early (n=12) | 35.8 (10.5) | 34.4 (8.5) | 0.51 |
| Non-early (n=10) | 36.0 (10.5) | 36.1 (8.5) | 0.97 |
| **Preconditioning** |  |  |  |
| MAC (n=15) | 36.8 (9.2) | 36.0 (10.1) | 0.69 |
| RIC (n=7) | 33.9 (10.8) | 33.4 (11.1) | 0.89 |
| **TBI** |  |  |  |
| ≤8 Gy (n=13) | 36.7 (10.5) | 34.4 (10.4) | 0.38 |
| > 8Gy (n=9) | 34.7 (8.4) | 36.0 (10.5) | 0.51 |
| **Acute GVHD** |  |  |  |
| < Grade II (n=13) | 37.2 (8.9) | 38.3 (10.0) | 0.55 |
| ≥ Grade II (n=9) | 33.9 (10.5) | 30.7 (9.2) | 0.28 |
| **HHV-6B reactivation** |  |  |  |
| Not higher-level reactivation (n=8) | 38.1 (7.1) | 35.8 (12.8) | 0.44 |
| Higher-level reactivation (n=14) | 34.6 (10.7) | 34.9 (8.9) | 0.88 |

*SD* standard deviation, *MAC* myeloablative conditioning, *RIC* reduced-intensity conditioning, *TBI* total body irradiation, *GVHD* graft versus host disease, *HHV-6B* human herpesvirus 6B.

^a^ paired t-test.
