# Supplementary material for: Effects of human herpesvirus 6B reactivation on cognitive function in cord blood transplant recipients: a prospective multicenter study
Source: Int J Hematol. 2024 Feb 26;119(4):432–41. doi: 10.1007/s12185-024-03714-2 (PMC10960775; doi:10.1007/s12185-024-03714-2)
Supplement: Supplementary file 5 — Supplementary file5 (DOCX 25 KB) [file 12185_2024_3714_MOESM5_ESM.docx]

**Supplementary Table 4. Scores for the FACT-BMT**

|  | **FACT-BMT trial outcome** | | | | |  | **FACT-G total score** | | | | |  | **FACT-BMT total score** | | | | |
| --- | --- | --- | --- | --- | --- | --- | --- | --- | --- | --- | --- | --- | --- | --- | --- | --- | --- |
|  | **Day 70** | ***P*^a^** |  | **1 year** | ***P*^a^** |  | **Day 70** | ***P*^a^** |  | **1 year** | ***P*^a^** |  | **Day 70** | ***P*^a^** |  | **1 year** | ***P*^a^** |
|  | mean (SD) |  |  | mean (SD) |  |  | mean (SD) |  |  | mean (SD) |  |  | mean (SD) |  |  | mean (SD) |  |
| **Total cases** (N=19) | 53.8 (11.8) |  |  | 64.0 (14.4) |  |  | 67.4 (13.5) |  |  | 72.4 (12.3) |  |  | 89.5 (16.4) |  |  | 97.9 (16.3) |  |
| **Age, years** |  |  |  |  |  |  |  |  |  |  |  |  |  |  |  |  |  |
| <55 (n=11) | 54.5 (11.6) | 0.38 |  | 66.8 (12.4) | 0.46 |  | 66.6 (14.2) | 0.97 |  | 73.9 (10.3) | 0.74 |  | 89.9 (17.2) | 0.71 |  | 100.7 (13.6) | 0.43 |
| ≥55 (n=8) | 53.0 (12.9) |  |  | 60.1 (16.8) |  |  | 68.6 (13.3) |  |  | 70.2 (15.1) |  |  | 88.9 (16.4) |  |  | 94.2 (19.7) |  |
| **Acute GVHD** |  |  |  |  |  |  |  |  |  |  |  |  |  |  |  |  |  |
| < Grade II (n=12) | 53.2 (14.8) | 0.73 |  | 67.2(14.9) | 0.14 |  | 67.2 (16.7) | 0.9 |  | 74.9 (13.5) | 0.19 |  | 89.7 (20.5) | 0.58 |  | 101.6 (17.4) | 0.18 |
| ≥ Grade II (n=7) | 55.0 (3.6) |  |  | 58.5 (12.5) |  |  | 67.7 (6.1) |  |  | 68.1 (9.3) |  |  | 89.2 (6.2) |  |  | 91.6 (12.7) |  |
| **Chronic GVHD** |  |  |  |  |  |  |  |  |  |  |  |  |  |  |  |  |  |
| None (n=11) | 52.4 (11.4) | 0.14 |  | 60.1 (17.4) | 0.25 |  | 66.8 (13.7) | 0.59 |  | 68.4 (13.4) | 0.15 |  | 87.7 (16.1) | 0.23 |  | 92.9 (18.4) | 0.17 |
| Limited or extensive (n=8) | 55.9 (12.8) |  |  | 69.4 (6.6) |  |  | 68.3 (14.1) |  |  | 77.9 (8.4) |  |  | 92 (17.7) |  |  | 104.9 (10.1) |  |
| **Relapse of underlying disease within 1 year after transplantation** |  |  |  |  |  |  |  |  |  |  |  |  |  |  |  |  |  |
| No (n=15) | 53.9 (12.3) | 0.88 |  | 65.7 (11.5) | 0.29 |  | 67.0 (13.6) | 0.96 |  | 74.1 (12.9) | 0.19 |  | 88.9 (17.0) | 0.96 |  | 100.7(15.4) | 0.18 |
| Yes (n=4) | 53.8 (11.6) |  |  | 57.5 (23.3) |  |  | 68.8 (15.1) |  |  | 66.0 (7.7) |  |  | 91.8 (16.0) |  |  | 87.5 (17.3) |  |
| **Peak HHV-6 DNA** |  |  |  |  |  |  |  |  |  |  |  |  |  |  |  |  |  |
| Not higher-level reactivation (n=7) | 56.7 (16.0) | 0.37 |  | 71.9 (9.8) | 0.18 |  | 68.7 (17.3) | 0.97 |  | 76.9 (7.7) | 0.25 |  | 92.0 (22.5) | 0.53 |  | 104.2 (9.8) | 0.27 |
| Higher-level reactivation (n=12) | 52.2 (9.0) |  |  | 59.4 (14.9) |  |  | 66.7 (11.6) |  |  | 69.7 (13.9) |  |  | 88.1 (12.6) |  |  | 94.3(18.5) |  |

*FACT-BMT* Functional Assessment of Cancer Therapy-Bone Marrow Transplant, *SD* standard deviation, *GVHD* graft versus host disease, *HHV-6* human herpesvirus 6.

**^a^** Mann–Whitney *U* test.
